# Trace Element-Augmented Titanium Implant With Targeted Angiogenesis and Enhanced Osseointegration in Osteoporotic Rats

**DOI:** 10.3389/fchem.2022.839062

**Published:** 2022-02-17

**Authors:** Ran Yan, Jinhua Li, Qianju Wu, Xiangkai Zhang, Longwei Hu, Yuwei Deng, Ruixue Jiang, Jin Wen, Xinquan Jiang

**Affiliations:** ^1^ Key Laboratory of Stomatology, Department of Prosthodontics, College of Stomatology, Shanghai Ninth People's Hospital, Shanghai Jiao Tong University School of Medicine, National Center for Stomatology; National Clinical Research Center for Oral Diseases, Shanghai Engineering Research Center of Advanced Dental Technology and Materials, Shanghai Jiao Tong University, Shanghai, China; ^2^ School of Medical Technology, Beijing Institute of Technology, Beijing, China; ^3^ Stomatological Hospital of Xiamen Medical College, Xiamen, China; ^4^ Department of Oral and Maxillofacial-Head and Neck Oncology, Shanghai Ninth People's Hospital, Shanghai Jiao Tong University School of Medicine, Shanghai, China

**Keywords:** strontium, osteoporosis, osseointegration, angiogenesis, implant surface modification

## Abstract

Deteriorated bone quality in osteoporosis challenges the success of implants, which are in urgent need for better early osseointegration as well as antibacterial property for long-term stability. As osteoporotic bone formation tangles with angiogenic clues, the relationship between osteogenesis and angiogenesis has been a novel therapy target for osteoporosis. However, few designs of implant coatings take the compromised osteoporotic angiogenic microenvironment into consideration. Here, we investigated the angiogenic effects of bioactive strontium ions of different doses in HUVECs only and in a co-culture system with BMSCs. A proper dose of strontium ions (0.2–1 mM) could enhance the secretion of VEGFA and Ang-1 in HUVECs as well as in the co-culture system with BMSCs, exhibiting potential to create an angiogenic microenvironment in the early stage that would be beneficial to osteogenesis. Based on the dose screening, we fabricated a bioactive titanium surface doped with zinc and different doses of strontium by plasma electrolytic oxidation (PEO), for the establishment of a microenvironment favoring osseointegration for osteoporosis. The dual bioactive elements augmented titanium surfaces induced robust osteogenic differentiation, and enhanced antimicrobial properties. Augmented titanium implant surfaces exhibited improved bone formation and bone–implant contact under comprehensive assessment of an *in vivo* bone–implant interface. In conclusion, zinc- and strontium-augmented titanium surface benefits the osseointegration in osteoporosis *via* promoting osteogenic differentiation, exerting antibacterial efficacy, and stimulating early angiogenesis.

**Graphical Abstract:**
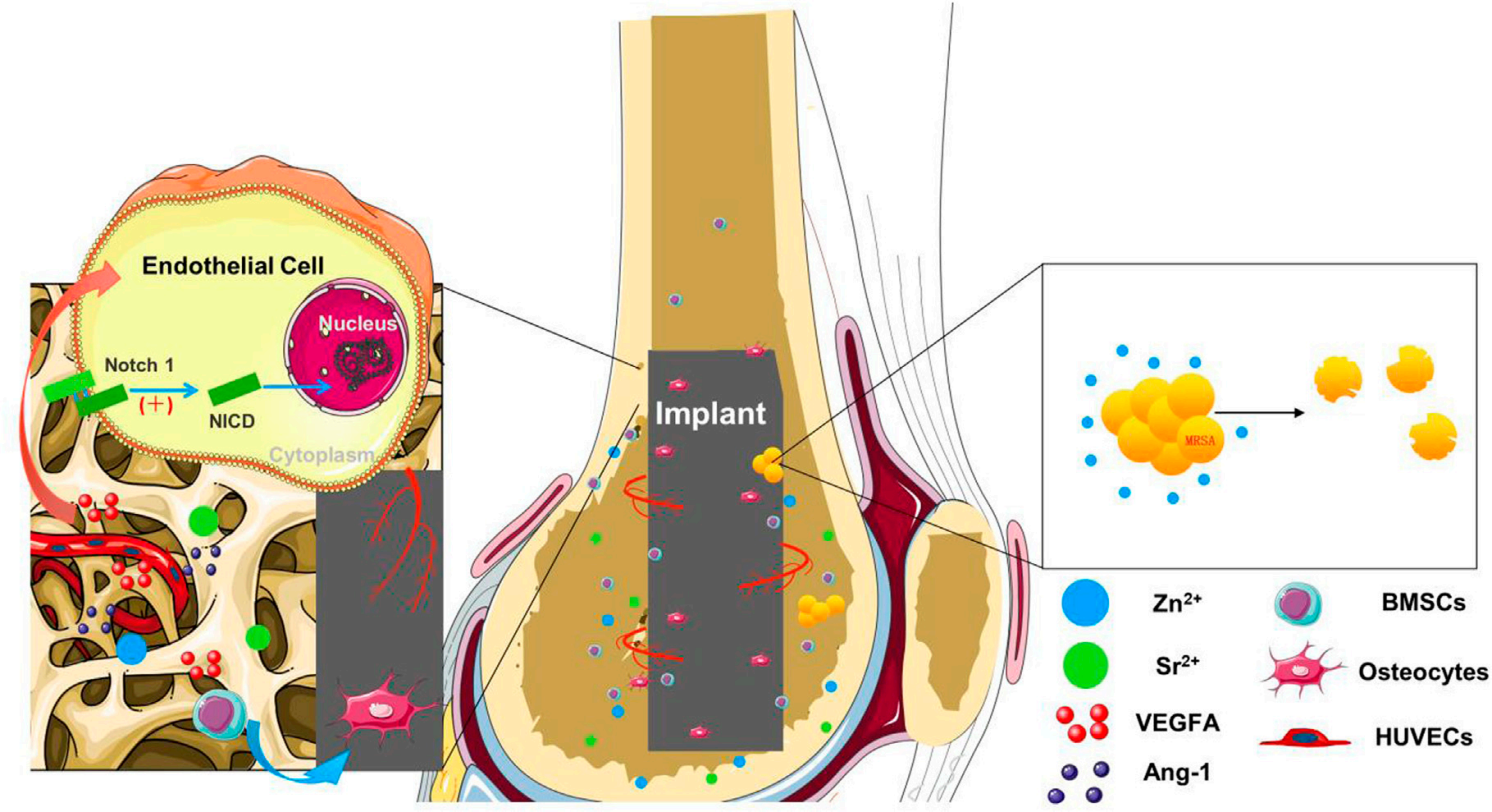


## Highlights

1) Osteoporotic bone implant niche with compromised angiogenesis and osteogenesis was targeted using hierarchical titanium surface with tailored trace elements.
2) Optimal strontium dose was screened by HUVECs only and a co-culture system with BMSCs mimicking angiogenesis and osteogenesis coupling.
3) Enhanced angiogenic, osteogenic, and antibacterial effects were promoted by Sr- and Zn-augmented bioactive surfaces for better osseointegration in osteoporotic rats.

## Introduction

With a large population and the fast growth of osteoporotic patients, the need for more favorable implants in dentistry and orthopedics is urgent, in that decreased bone mass and deteriorated bone microarchitecture hinder the success of osseointegration at the early stage (Dereka et al., 2018; Liu et al., 2021b). The ideal implant of osteoporosis is expected to exert osteogenic effects during the healing process, which is a complex matter involving angiogenesis (Irandoust and Müftü, 2020). Blood vessels contribute to bone metabolic microenvironment with access to nutrients and oxygen (Fu et al., 2020). Vascularization of ossifying tissue is highly required for bone growth and development while osteoporosis exhibits vascular defects with reduced connection between osteogenesis and angiogenesis (Song et al., 2019; Tong et al., 2019). Studies have found that reduction in vascular flow in the lower extremities has a close correlation with decreased bone mineral density (Vogt et al., 1997). Therefore, improvement of bone angiogenesis has won widespread attention as a novel target in the prevention and treatment of osteoporosis (Sivaraj and Adams, 2016; Fu et al., 2020; Chen et al., 2021). Implants with the function of promoting angiogenesis have potential to create a desirable osseointegration environment in osteoporotic bone (Zhou et al., 2020). It is promising to promote the osteoporotic bone implant niche with compromised angiogenesis and osteogenesis using hierarchical titanium surface with tailored trace elements.

Strontium (Sr), one of the most potent bioactive element candidates applied in implant surface modification, conducts a dual action of stimulating the differentiation of osteoblast cells and inhibiting maturation and activity of osteoclasts (Querido et al., 2016; Pilmane et al., 2017; Wu et al., 2020). A large amount of clinical and experimental evidence support the idea that Sr ions improve bone microarchitecture for the prevention or treatment of osteoporosis (Yang et al., 2015; Maria et al., 2017; Martiniakova et al., 2020). Recent attention has been paid to the role of Sr in supporting bone angiogenesis. Sr-incorporated materials have been proven to enhance bone mesenchymal cell (BMSC) angiogenic differentiation and result in vascular endothelial growth factor (VEGF) secretion (Gu et al., 2014; Wei et al., 2019). Previous studies have found that materials containing Sr exhibited potential of promoting angiogenesis. However, it remains unknown how Sr functions on the cell lineages related to angiogenesis. Endothelial cells (ECs) secrete angiogenic factors and control bone angiogenesis, but the underlying action of Sr on ECs was seldom explored (Langen et al., 2017; Chiesa et al., 2020). Besides, successful angiogenesis is a staged process constituting sprouting, maturation, and remodeling, which is regulated by a complex signaling network of various cytokines (Augustin et al., 2009). Previous studies have found that VEGF acts early to initiate angiogenesis, whereas Ang-1 acts later to induce vessel maturation (Saharinen et al., 2011). Designed timing VEGF and Ang-1 gene delivery to mimic endogenous angiogenesis resulted in sustained and functional neovascularization (Smith et al., 2012). Hence, it is necessary to further assess the effects of Sr on different angiogenic stages and to investigate the optimal dose of Sr in promoting angiogenesis.

In this study, the effects of Sr on ECs were explored in an only-human umbilical vein endothelial cells (HUVECs) system and a co-culture system with BMSCs. This is the first time to screen concentration of Sr in a dual environment of bone osteogenesis and angiogenesis, which aims to better mimic the complex healing process *in vitro* for more comprehensive investigation of effective doses of Sr. It is expected to provide reference for the underlying mechanisms of metal ions involved in vascular bone regeneration. In our study, the dose of Sr utilized to modify a titanium implant is based on the results of *in vitro* screening. The modification of titanium implant surface is through plasma electrolytic oxidation (PEO) with tailored trace elements, Sr and zinc (Zn). PEO is a facile, controllable, and cost-effective surface modification method of implants. It can dope multiple types and desired doses of elements to the implant surface, which endow implants biological functions and micro–nano surface structures (Wang et al., 2019; Zhao et al., 2019; Cao et al., 2021). Zn minimizes several bacterial and fungal strains’ adherence to titanium substrates with better biosafety than copper and argentum (Vimbela et al., 2017). The antibacterial poverty of Zn benefits osseointegration and stability of implants in the long term for osteoporotic patients inclined to undergo faster and more severe inflammation development such as peri-implantitis (Davies, 2003). The dual element-augmented Ti surfaces with Sr in different doses as well as Zn were tailored to assess the osteogenic effects of BMSCs of ovariectomized (OVX) rats *in vitro* and were implanted into the rat model to determine whether osseointegration can be potentially improved *in vivo*.

## Material and Methods

### Sample Preparation

Metallic titanium foils (purity >99.85 wt%) with dimensions of 10 mm × 10 mm × 1 mm or 20 mm × 20 mm × 1 mm were ground, polished to a shiny surface texture, and then ultrasonically cleaned with ethanol and ultrapure water several times to obtain a clean and homogeneous surface, followed by drying. In the animal experiment, Ti rods (purity >99.85 wt%) with a diameter of 2 mm and a length of 7 mm were used. TiO_2_ coatings were prepared on titanium substrate by PEO in calcium/phosphate-containing electrolyte with calcium acetate (C_4_H_6_O_4_Ca·H_2_O) and glycerophosphate disodium salt (C_3_H_7_Na_2_O_6_P·5H_2_O, 50 mM). Zn and Sr elements were introduced by adding zinc acetate (C_4_H_6_O_4_Zn·2H_2_O, 60 mM) and strontium acetate (C_4_H_6_O_4_Sr·1/2H_2_O) to electrolyte. The total concentration of C_4_H_6_O_4_Ca·H_2_O and C_4_H_6_O_4_Sr·1/2H_2_O was constant at 100 mM, and the molar ratios of Sr:(Sr + Ca) were adjusted to 0:4, 1:4, 2:4, and 3:4, respectively. The prepared samples were designated as TiO_2_, Zn@TiO_2_, 1SrZn@TiO_2_, 2SrZn@TiO_2_, and 3SrZn@TiO_2_, respectively.

### Surface Characterization

The surface morphology and cross section were characterized by field-emission scanning electron microscopy (SEM; Hitachi SU8010) equipped with an energy-dispersive x-ray spectrometer (EDS). The crystallinity of the coatings was determined using an x-ray diffractometer (XRD; Rigaku Ultima IV) fitted with a Cu K*α* (*λ* = 1.541 Å) source. Phase identification was carried out with the help of the standard JCPDS database. The elemental chemical states of samples were investigated by x-ray photoelectron spectroscopy (XPS; ESCALAB 250Xi) with a Mg K*α* (1,253.6 eV) source.

### Ion Release Measurement

All the samples were soaked in 10 ml of Dulbecco’s Modified Eagle’s medium (DMEM, Gibco, United States) at 36.5°C for 1, 4, 7, and 14 days successively. At the end of each incubation point, the leaching liquid was collected and the release amounts of Ca, P, Sr, and Zn ions were determined by inductively coupled plasma mass spectrometry (ICP-MS; Agilent 7,800).

### Antibacterial Test

The antibacterial property of zinc-doped samples was evaluated by using bacterial counting method using methicillin-resistant *Staphylococcus aureus* (MRSA, ATCC 43300). After sterilization, a droplet of bacteria solution at a concentration of 10^7^ CFU/ml in trypticase soy broth (TSB) was introduced onto each sample to a density of 60 μl/cm^2^. The samples with bacteria solution were incubated at 37°C for 24 h. For SEM observation, those samples were then fixed and dehydrated in a series of ethanol solutions (30, 50, 75, 90, 95, and 100 v/v%) for 10 min each sequentially.

For the spread plate evaluation, the bacteria solution was dissociated from surface and then cultivated on standard agar culture medium at 37°C for 24 h. Finally, the numbers of live bacteria were counted according to the National Standard of China GB/T 4789.2 protocol, and the antibacterial ratio (*R*) was calculated by the formula, *R* = (A−B)/A×100%: A, the average number of bacteria on control (CFU/sample); B, the average number of bacteria on testing sample (CFU/sample).

The Live/Dead BacLight Bacterial Viability Kit (Invitrogen, United States) was used to investigate the antibacterial properties of titanium samples. *Porphyromonas gingivalis* (*P. gingivalis*) suspension was inoculated onto the samples and cultured overnight. Then, bacteria on samples were dyed with a 1:1 mixture of SYTO 9 and PI for 15 min in the darkness and observed through a confocal laser-scanning microscope (CLSM; Leica TCS Sp^2^ AOBS, Germany).

### Cell Culturing

The cells used in this study were incubated in a humidified atmosphere of 5% CO_2_ at 37°C. HUVECs were purchased from ScienCell and human BMSCs (hBMSCs) from the American Type Culture Collection (ATCC). Endothelial Cell Medium (ECM) (Sciencell, United States), containing 10% fetal bovine serum (FBS), 100 U ml^−1^ penicillin, 100× endothelial cell growth supplement (ECGS), and 100 U ml^−1^ streptomycin (both from Sciencell, United States), was used for cultivation and expansion of HUVECs. Human BMSCs were cultured in minimum essential medium *α* medium (*α*-MEM) (Gibco, United States) supplemented with 10% FBS (Gibco, United States). Cells at passages 3 to 6 were used in our experiment. The co-culture system is constructed by a transwell chamber (6-well, diameter 4.0 μm) (Corning, United States) with HUVECs in the upper chamber and hBMSCs in the lower chamber. The co-culture medium is mixed by ECM and *α*-MEM at ratio of 1:1.

The Animal Committee of the Ninth People’s Hospital Affiliated to Shanghai Jiao Tong University School of Medicine approved all experimental protocols concerning animals. We adopted the generally accepted method for ovariectomy operation (Watanabe et al., 2020; Liu et al., 2021a). Briefly, ovariectomy was performed on 3-month-old female SD rats through two dorsal incisions that were made in each rat. Three months after the operation, the OVX rats were sacrificed, and the metaphysis from both ends of the femurs were cut off to enable the marrow to be flushed out. The cells were then cultured in DMEM (GIBCO Laboratories, Grand Island, NY) in 10-cm-diameter plates for nearly 2 weeks at 37°C in a 5% CO_2_ incubator. It takes approximately 6–7 days to obtain primary OVX rat-derived BMSCs (OVX BMSCs). The cultured cells from passage 2 were used for further studies.

### Cell Proliferation Assay

The cell proliferation activity assay of the OVX BMSCs on different samples was evaluated by the Cell Counting Kit-8 (CCK-8, Dojindo Laboratories Inc., Kumamoto, Japan) test. Initially, 2.0 × 10^4^ cells/ml were seeded onto each flat sample in a 24-well plate for 1, 4, and 7 days of culture. At each time point, after incubation for 1 h, the DMEM-CCK8 solution was carefully moved to a 96-well plate, and absorbance was read at the wavelength of 450 nm by a microplate spectrophotometer (Benchmark Plus, Tacoma, Washington, United States). All experiments were performed in triplicate, and the results were analyzed by plotting cell growth curves according to the absorbance readings. All experiments were performed in triplicate.

### Alkaline Phosphatase Activity Assay

After being cultured for 7 days in DMEM, cells seeded on different samples were stained or collected, lysed, and incubated with p-nitrophenyl phosphate (pNPP) (Sigma, St. Louis, MO, United States) at 37°C for 30 min and ALP activity was detected by the measurement of optical density (OD) values at 405 nm, while total protein content was measured with the Bradford method at 630 nm of OD values according to a series of BSA (Sigma, United States) standards. Finally, ALP activity levels were normalized to the total protein content and expressed as OD values at 405 nm/mg of total cellular proteins. All the measurements were made in triplicate.

### Quantitative Real-Time PCR Assay

At the time point of 4 and 7 days, cells seeded on each flat sample were collected and suspended in TRIzol reagent (Invitrogen, United States), and the total RNA was harvested to synthesize complementary DNA using a PrimeScript 1st Strand cDNA Synthesis kit (Takara, Japan) according to the manufacturer’s instructions. The expression of key osteogenic differentiation markers osteocalcin (*Ocn*), Osteopontin (*Opn*), bone morphogenetic protein 2 (*Bmp2*), and alkaline phosphatase (*Alp*) and angiogenic markers (*VEGFA, Ang-1*) were measured by using reverse transcription polymerase chain reaction (RT-PCR) with Bio-Rad MyiQ single color Real-time PCR system, while the housekeeping genes, *β-actin* and *Gapdh*, were used for normalization. Purified gene-specific primers above were synthesized commercially (Sangon, Co. Ltd., Shanghai, China) and the primer sequences used in present study are listed in Supplementary Table S1. All experiments were performed in triplicate to obtain the average data.

### Immunofluorescence

The OVX BMSCs were seeded on titanium samples at a density of 5 × 10^5^ cells/ml for 4 days to detect the OCN expression, and then the samples were washed with PBS three times and fixed in 4% paraformaldehyde for 30 min. The cells were then permeabilized with 0.1% Triton X-100 for 30 min and blocked in 10% goat serum for 1 h at room temperature. A specific primary antibody targeting osteocalcin (Abcam, United States) was added at 1:100 dilutions and co-incubated overnight at 4°C. TRITC-conjugated anti-mouse IgG antibody (Boster, CHINA) at 1:100 dilutions was used in the dark. The specimens were observed using a CLSM (Leica TCS Sp^2^ AOBS, Germany) while cellular nuclei were contrast-labeled with DAPI (Sigma, United States).

### Western Blot

For the Western blot assay, the OVX BMSCs were seeded on five samples in 6-well plates at a density of 2 × 10^5^ cells/well and cultured for 7 and 10 days while HUVECs were seeded in 6-well plates with ECM containing 0, 0.2, 1, and 5 mM SrCl_2_ and cultured for 3 and 7 days. After being collected from sample surfaces, cells were lysed with a protein extraction reagent containing protease inhibitor cocktail, phosphatase inhibitor cocktail, and phenylmethanesulfonyl fluoride (PMSF) (Kangchen, China). The obtained protein concentration was measured using a Bio-Rad protein assay kit. Then, equal amounts of protein from different samples were separated on SDS-polyacrylamide gel electrophoresis (PAGE) and electro-transferred to a polyvinylidene difluoride membrane (PVDF, Pall, United States). Membranes were incubated with specific primary antibodies VEGFA (sc-7269, United States, dilution, 1:1,000) overnight at 4°C. Finally, the membranes were visualized using horseradish peroxidase (HRP)-conjugated rabbit anti-mouse (Beyotime, China) using the ECL plus reagents (Amersham Pharmacia Biotech, United States) by a UVItec ALLIANCE 4.7 gel imaging system.

### Surgical Procedures

In this study, a rat femoral model was used, and the surgical procedures were conducted as described previously (Wen et al., 2015). The rats were anesthetized by intraperitoneal injection of ketamine. After their hind limbs were shaved, a 10-mm longitudinal incision was made across the knee joint along the lateral side of the extensor mechanism. Using a rotary drill, a cylindrical hole measuring approximately 2.2 mm in diameter was created along the long axis of the femur. Two implants were randomly and bilaterally placed into each rat. The soft tissues were sutured after relocation of the patella and reconstruction of the extensor mechanism. Twenty rats were operated on in this study, and they were randomly divided into 5 groups.

### Sequential Fluorescent Labeling and Sample Preparation

The process of new bone formation and mineralization was assessed by polychrome sequential fluorescent labeling method. At 2, 4, and 6 weeks after surgery, 30 mg/kg alizarin red (Sigma) and 20 mg/kg calcein (Sigma) were intraperitoneally administered respectively. The 20 animals that underwent surgery were randomly allocated into 5 groups. Each observation group included 4 rats, and both legs from each rat were assessed, for a total of 8 legs per group (*n* = 8). All rats were sacrificed at 8 weeks after surgery. Following this, 16 femurs were harvested and trimmed into smaller blocks.

### Micro-CT Assay

The presence of newly formed bone around the implants was detected by Micro-CT (GE explore Locus SP Micro-CT, United States). The parameters of scanning were set at 80 kV with an exposure time of 3,000 ms and a resolution of 15 µm. Three-dimensional images were reconstructed using NRecon software (SkyScan, United States) and a CTvol program (SkyScan). The bone volume fraction (bone volume/total volume, BV/TV) and trabecular bone thickness (Tb.Th) were determined for newly grown bone tissues using DataViewer software (SkyScan) and a CTAn program (SkyScan).

### Histological and Histomorphometric Observation

To quantitate the bone formation and mineralization in the raised area, sections were observed for fluorescence labeling CA under Leica CLSM. Excitation/emission wavelengths for each of the fluorescence were as follows: 488/517 nm (CA, green); it represented the bone formation and mineralization at 6 weeks after the operation. The sections were further stained with van Gieson’s picrofuchsin for histological observation and histomorphometric analysis after fluorescent analysis.

### Push-Out Test

In order to test the bone bonding strength of implants, three specimens of bilateral femurs per group were performed for the biomechanical test by using the universal material testing system (Instron, High Wycombe, United Kingdom). A special holder was designed to fix all test samples to ensure the test force is parallel with the long axis of implants. The peak load values were used to evaluate the failure load with a 5 mm/min loading rate of test force.

### Enzyme Linked Immunosorbent Assay

Concentrations of VEGFA and Ang-1in co-culture media were determined by the ELISA technique using a human anti-VEGFA, anti-Ang-1 assay kit (RayBiotech, United States). To detect the secretion of the HUVECs–hBMSCs co-culture system, HUVECs and hBMSCs were incubated in 6-transwell plates at 37°C with co-culture medium. After the corresponding treatment, the co-culture medium was collected and stored at −80°C. One hundred milliliters of the sample and standard solution was added into the 96-well plate and incubated overnight at 4°C. The plate was washed four times with washing buffer and then the anti-human VEGFA antibody or anti-human Ang-1 antibody was added to incubate for 2 h at 4°C. After washing four times again, 100 ml of streptavidin horseradish peroxidase reagent was added and incubated for 30 min. After washing three times, 100 ml of TMB substrate solution was added to the wells and incubated for 15 min in the dark at 37°C. Finally, the stop solution was added to terminate the reaction. The spectrophotometer (Bio-Tek) was used to determine VEGFA or Ang-1 concentration at 450 nm wavelength.

Proteins of HUVECs after stimulation by 1 mM Sr for 1 h and the control were prepared and ELISA (Total Notch1 cat. No. 7245; Cleaved Notch1 cat. No. 7194; Cell Signaling, Beverly, United States) was performed according to the manufacturer’s protocols. Colorimetric readings were made at 450 nm on spectrophotometer (Bio-Tek).

### Tube Formation Assay

All the plates and tips were pre-cooled before coating Matrigel Matrix (Corning, United States). The 48-well plates were coated with Matrigel (150 μl/well) and put in a humidified atmosphere for at least 30 min at 37°C. Then, trypsin-harvested HUVECs were suspended in ECM (Sciencell, United States) and seeded onto the plated Matrigel (2 × 10^5^ cells per well). After HUVECs attached, medium was changed to ECM with 0, 0.2, 1, and 5 mM SrCl_2_, respectively, or changed to co-culturing medium collected at day 7 while testing the angiogenic poverty of the co-culture system.

### Statistical Analysis

All data were expressed as means ± standard deviation. Statistical significance was assessed for the above assays performed by ANOVA and SNK post hoc or Kruskal–Wallis nonparametric procedure followed by Mann–Whitney *U* test for multiple comparisons based on the normal distribution and equal variance assumption test, using SPSS v.10.1 software (IBM SPSS, Armonk, New York, United States).

## Results and Discussion

### Strontium Stimulates the Osteogenic Ability of Human Umbilical Vein Endothelial Cells and Angiogenic Ability of Human Umbilical Vein Endothelial Cells *in vitro*

Bone vessels create a microenvironment supporting the survival and differentiation of osteoblasts (Diomede et al., 2020). To identify the role of Sr in the microenvironment around implant sites, it is of significance to first test the direct effects of Sr on osteogenesis and angiogenesis, respectively.

**FIGURE 1:**
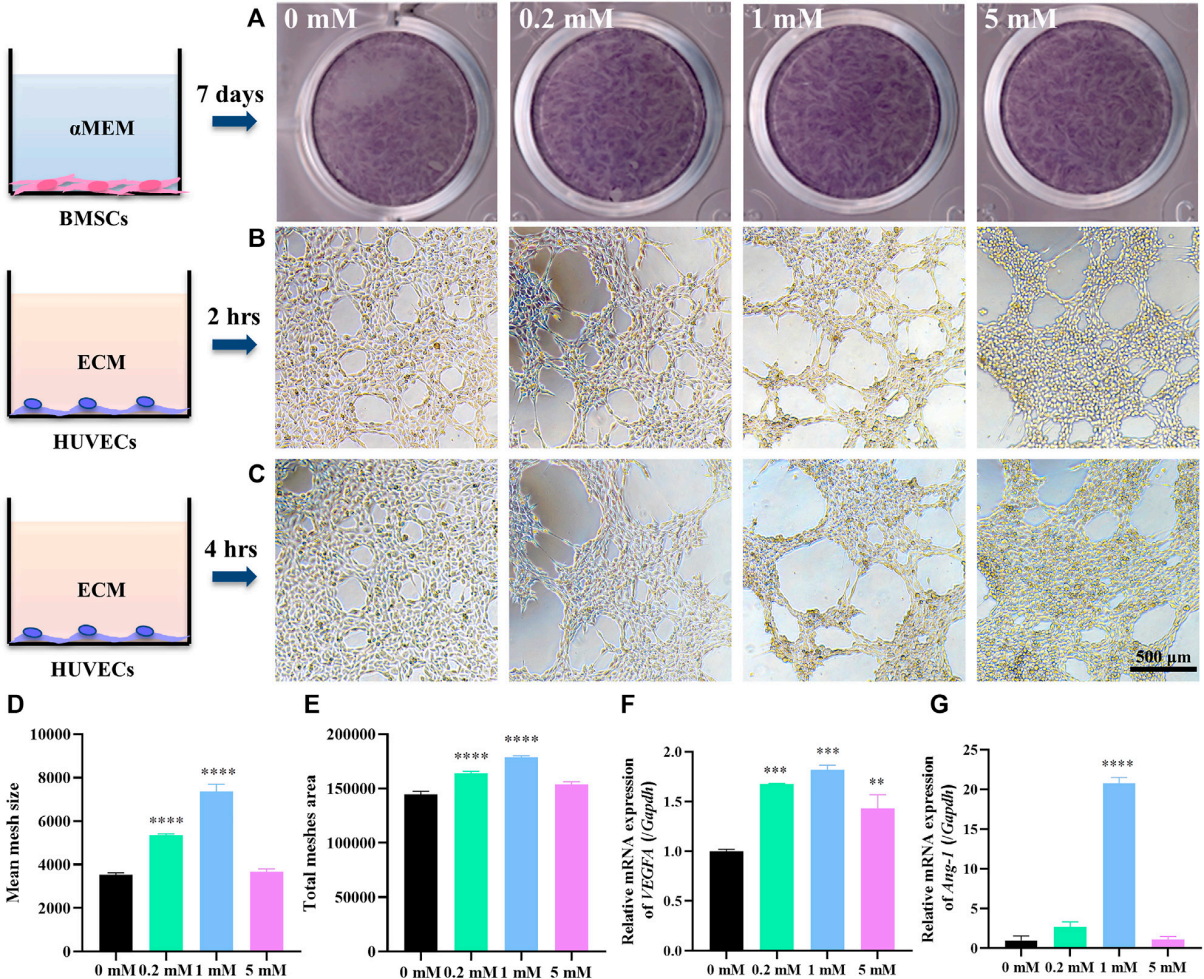
Sr stimulates the osteogenic ability of hBMSCs and angiogenic ability of HUVECs *in vitro*. **(A)** ALP staining of hBMSCs cultured in *α*-MEM with Sr at 0, 0.2, 1, and 5 mM for 7 days. **(B,C)** Tube formation assay of HUVECs cultured in ECM with SrCl_2_ at 0, 0.2, 1, and 5 mM for 2 h **(B)** and 4 h **(C)** with quantitative analysis by ImageJ of **(D)** mean size of meshes and **(E)** total mesh area at time point “2 h”. **(F)** Relative expression of VEGFA gene in HUVECs cultured for 3 days and **(G)** Ang-1 gene for 7 days in ECM with Sr at 0, 0.2, 1, and 5 mM (*n* = 3, *p < 0.05, **p < 0.01, ***p < 0.001, ****p < 0.0001, compared with the 0 mM group).

To evaluate the osteogenic effects of Sr, hBMSCs were cultivated in *α*-MEM containing different doses of Sr for 7 days and tested ALP expression as an early biomarker of osteogenesis through ALP staining. Figure 1A shows that ALP expression increased in groups containing Sr. Current studies demonstrate that the actual effective concentration of Sr fluctuates depending on different implanting materials. The effective Sr concentration on osteogenesis of Sr-containing bioceramics is 2–6 μg/ml (Barrioni et al., 2019) while that of biodegradable Zn–Sr binary alloy is 0.25–0.5 μg/ml (Jia et al., 2021). In this study, 0.2–5 mM Sr promoted osteogenic differentiation.

Few studies screen the effects of different doses of Sr on angiogenesis. Thus, the angiogenic effects of Sr on HUVECs were detected by tube formation assay. Tube formation assay is a conventional approach to evaluating angiogenic ability of ECs *in vitro* by measuring the tube-like structures formed on an extracellular matrix, Matrigel (Lee and Kang, 2018). In this study, HUVECs were plated on Matrigel and cultured with ECM containing different doses of Sr. Figures 1B,C present the meshed networks of HUVECs formed on Matrigel. HUVECs with 1 mM Sr for 2 h in Figure 1B formed larger lumens as approved by ImageJ analysis where 1 mM Sr group showed largest mean size of meshes in Figure 1D and most total mesh area in Figure 1E (Carpentier et al., 2020). Figure 1C shows that tube structures tended to enlarge after 4 h of Sr stimulation, especially in the 0.2 and 1 mM group compared with 2-h images in Figure 1B. It is noted that the 5 mM group presented cell aggregation on the edge of the lumens, which was an indication of angiogenic regression. It suggests the dual effects of Sr on ECs: although Sr promotes angiogenesis for the formation of new lumens, the tubes could collapse quickly if the vessel maturation did not happen in time. Therefore, it is important to balance the time of vessel formation and maturation by administering the proper dose of Sr.

To further clarify the effects of strontium in vessel formation and maturation, HUVECs were cultivated in ECM containing Sr for checking days. Gene expression of VEGFA as an early angiogenic marker at an early stage was detected at day 3 and that of Ang-1 as a biomarker of vessel maturation was explored at a later stage, day 7. PCR analysis showed that Sr promoted gene expression of VEGFA especially in the 1 mM group at day 3 (Figure 1F). The expression of Ang-1 gene was strong in the 0.2 and 1 mM groups at day 7 (Figure 1G). VEGFA as a crucial regulator of both normal and pathological angiogenesis binds to receptors on ECs to stimulate proliferation and cell migration (Ferrara and Adamis, 2016; Melincovici et al., 2018). At the initial stage of sprouting angiogenesis, VEGFA released from injured ECs increases vascular permeability contributing to the migration of ECs for the initiation of tubulogenesis (Melincovici et al., 2018). Maintenance of the resting EC phenotype requires tight association with peri-ECs. However, at some time during vessel maturation and remodeling, the survival of ECs becomes independent of VEGFA. Vascular immaturity leads to excessive permeability, poor perfusion, and increased hypoxia. It is thus of significance to release factors that recruit perivascular cells and promote stabilization. Angiopoietins (Ang) have been shown to be required for vessel stabilization *via* interaction with perivascular cells. Ang-1 is predominantly released by perivascular cells and activates the Tie2 receptor present on ECs (Viallard and Larrivee, 2017). Ang–Tie signaling functions as a key regulator of adult vascular homeostasis, which induces cell–cell contacts and transduces survival signals to inhibit paracellular permeability (Augustin et al., 2009; Melincovici et al., 2018). Thus, the lower gene expression of Ang-1 in the 5 mM Sr group may result in reduced vessel stability compared with the 0.2 and 1 mM group as shown in tube formation assay (Figure 1C) (Fagiani and Christofori, 2013). The results in this study indicate that 0.2–1 mM Sr has potential to stimulate VEGFA expression at early stage of angiogenesis and to maintain vessel stability through Ang-1 induced pericyte recruitment.

### Strontium Stimulates the Angiogenesis Ability of Human Umbilical Vein Endothelial Cells in the HUVECs–hBMSCs Co-culture System

**FIGURE 2:**
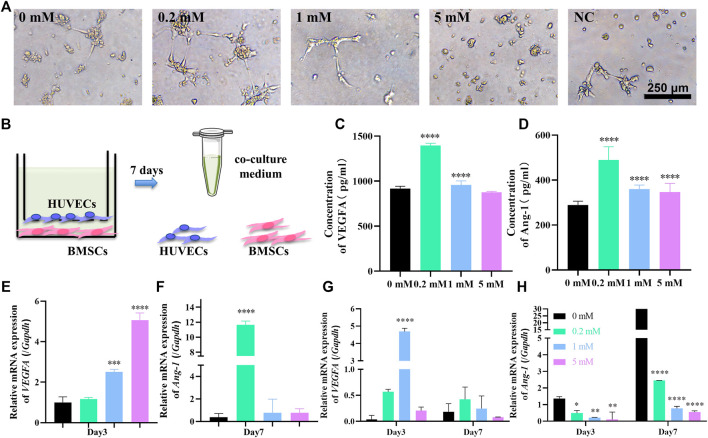
Sr stimulates the angiogenesis ability of HUVECs in the HUVECs–hBMSCs co-culture system. **(A)** Tube formation assay for 3 h with medium containing 0, 0.2, 1, and 5 mM Sr collected at day 7 in the co-culture system and fresh co-culture medium without Sr as negative control. **(B)** The illustration of the co-culture system consisting of hBMSCs at the lower chamber and HUVECs at the upper chamber using a 1:1 mixture of *α*-MEM and ECM as co-culture medium; at day 7, the co-culture medium was collected for tube formation assay and ELISA while the hBMSCs and HUVECs were collected for PCR assay. **(C,D)** ELISA of VEGFA **(C)** and Ang-1 **(D)** in co-culture medium at day 7. **(E)** Relative expression of VEGFA gene of HUVECs cultured for 3 days and **(F)** relative expression of Ang-1 gene of HUVECs cultured for 7 days in the co-culture system. **(G,H)** Relative expression of VEGFA **(G)** and Ang-1 **(H)** genes in hBMSCs cultured for 3 and 7 days in the co-culture system (*n* = 3, *p < 0.05, **p < 0.01, ***p < 0.001, ****p < 0.0001, compared with the 0 mM group).

Strontium-doped implants are located in a dual environment of bone and blood vessels. For the simulation of the osteogenesis and angiogenesis environment *in vivo* after implantation, we used the transwell co-culture model, with HUVECs in the upper compartment and hBMSCs in the lower compartment, establishing the HUVECs–hBMSCs co-culture system for the first time to study the role of Sr in the osteogenesis and angiogenesis environment (Figure 2B). Although the effects may be stronger in a direct contact system, the design of an indirect and simultaneous dual-stimulating model can better simulate an *in vivo* environment where capillary ECs survive through the trabecular bone and bone mesenchymal cells in bone matrix with limited direct contact between different cell types, mainly *via* paracrine factors.

To test the angiogenic potential of the co-culture environment, the medium of the system was collected at day 7 and was tested in tube formation assay (Figure 2A). Compared to the fresh co-culture medium as negative control, the inclination of tube formation was obvious in the medium from the system with Sr concentration of 0, 0.2, and 1 mM. Although without the support of complete ECM, HUVECs stretched and extended to form branches and loops especially in the co-cultured 0.2 mM Sr group (Figure 2A). There is no trace of tube formation in the 5 mM group, which indicates that overdose of Sr may affect the angiogenesis of HUVECs, consistent with the result in the only-HUVECs system (Figures 1C, 2A).

The angiogenic potential of co-culture medium is related to the secretion of angiogenic factors by hBMSCs and HUVECs. ELISA was thus performed to test the concentration of VEGFA and Ang-1 in the co-culture medium at day 7. Figure 2C shows that the concentration of VEGFA in the co-culture medium is significantly increased under the effect of 0.2 mM Sr at day 7; 0.2 mM is also the peak concentration of Ang-1 in Figure 2D. The concentration of the factors in co-culture media is determined by the secretion of both HUVECs and hBMSCs. To verify the contribution of each cell type, PCR assay was conducted to evaluate the gene expression of VEGFA and Ang-1 in HUVECs and BMSCs, respectively. VEGF gene expression of HUVECs started to increase in the co-culture system in 0.2 mM at day 3 (Figure 2E). VEGFA of hBMSCs also increased at the early stage with the strongest gene expression in 1 mM Sr at day 3 while the gene expression weakened at day 7 (Figure 2G). Figure 2F shows that Ang-1 gene expression significantly increased in the 0.2 mM Sr group at day 7, while that of hBMSCs decreased in Sr-groups (Figure 2H).

The results of the co-culture system show that 0.2 mM Sr stimulates the peak concentration of VEGFA and Ang-1 in the co-culture system, while in the only-HUVECs system, the most effective concentration was 1 mM. This suggests that the creation of an angiogenic environment in a co-culture system requires a lower dose of Sr than in a only-HUVECs system. It is hypothesized that lower Sr stimulates comprehensive functions of multiple cell lineages surrounding implant sites. In the co-culture system, Sr promotes the VEGFA expression at an early stage and enhances the Ang-1 expression of HUVECs later. The expression of VEGFA of hBMSCs also increased under the effects of Sr while that of Ang-1 decreased. Since Sr contributes to osteogenic differentiation of hBMSCs shown in Figure 1A, it is supposed that hBMSCs could upregulate secretion of VEGFA while reducing the secretion of Ang-1 during osteogenesis for the rapid growth of new vessels at an early stage (Chiesa et al., 2020).

The results in this study suggest that Sr can promote not only osteogenesis but also angiogenesis. However, there remain questions to be answered further as the healing process after implantation has been complex and constantly changing. The cells involved in the mechanism of osteo-angiogenesis coupling and the communication mechanism behind it still need further understanding because the HUVECs–hBMSCs co-culture model reflects part of the implanting situation. The immune system also plays an important role in the healing process with close interaction between osteogenesis and angiogenesis. Miya Kang et al. found that the recruitment of monocytes/macrophages is crucial in the early stage of osseointegration *via* exosomes (Kang et al., 2020). Guo et al. revealed that RAW264.7 cells enhanced the ability of tubule formation and migration of HUVECs and their differentiation into pro-osteogenesis vessels (Guo et al., 2020). The immune system is supposed to be another vital role in the communication of osteogenesis and angiogenesis. In addition, Xie et al. found that preosteoclasts are involved in the coupling by secreting angiogenic factors (Xie et al., 2014). The exact cell communication patterns involved in Sr coupling and how to promote angiogenesis with facilitated osteogenesis still need to be elucidated in future studies.

### Materials Characterization

With the screening of Sr effects on HUVECs *in vitro*, we tailored titanium with strontium for better osseointegration. As Zn has been a conventional antibacterial element, the tailored titanium was also modified with Zn for the prevention of implantitis. According to the molar ratios of Sr:(Sr + Ca) in titanium coating 0:4, 1:4, 2:4, and 3:4, the prepared samples were designated as TiO_2_, Zn@TiO_2_, 1SrZn@TiO_2_, 2SrZn@TiO_2_, and 3SrZn@TiO_2_, respectively.

**FIGURE 3:**
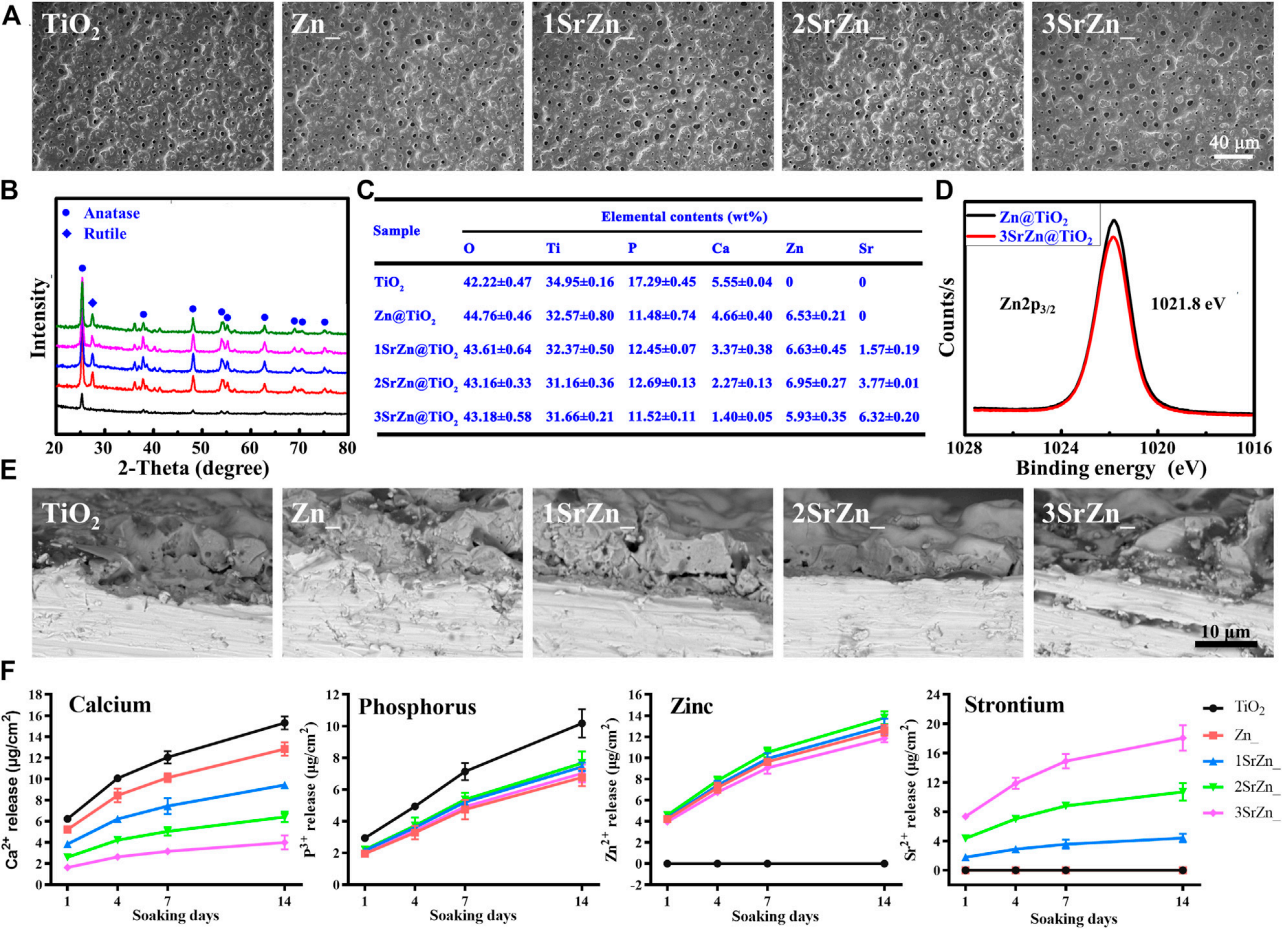
Materials characterization. **(A)** SEM images of the surface microstructure of pure TiO_2_ and Zn and/or Sr-doped TiO_2_ samples after PEO treatment. **(B)** XRD spectra of the samples. **(C)** EDS mapping of the major elements in the coatings of the samples. **(D)** XPS spectra of Zn2p_3/2_. **(E)** SEM images showing the cross section of the samples. **(F)** ICP-MS determination of Ca, P, Sr, and Zn ions released from samples after 1, 4, 7, and 14 days.

Homogeneous porous surface micro/nano topographies were prepared on titanium substrate, shown by SEM (Figure 3A). The processing method, PEO, can create a uniform ceramic oxide film *in situ* on a complex surface. It was noted that all the samples maintained a similar micro–nano surface structure, indicating that the incorporation of elements did not significantly alter the surface morphology of coatings. Modified implant surfaces have been found to alter bone turnover in osteoporotic patients to stimulate functional osseointegration (Lotz et al., 2020). Micro–nano porous structures provide space for nutrition, vascularization, and finally bone ingrowth and implant fixation for the balance of load transfer (Gorgin Karaji et al., 2017; He et al., 2020). With regard to the phase composition, XRD shows that the surface coating was mainly composed of anatase TiO_2_ before treatment while attributive diffraction peaks of rutile phase also appeared after zinc doping (Figure 3B), which suggests that zinc content have influence over the nanocrystalline structure of the coatings. EDS analysis presents that elements of Sr and Zn entered into the TiO_2_ crystal lattice (Figure 3C). According to the Zn2p_3/2_ XPS spectra (Figure 3D), the binding energy at 1,021.8 eV was assigned to the Zn2p in ZnO. This indicates that the doped zinc existed in the form of ZnO. The SEM showed that the coatings of 5 groups have a layer thickness of 8–10 μm (Figure 3E).

The release profiles of Ca, P, Zn, and Sr ions from the sample surfaces after soaking in 10 ml of DMEM within 14 days (Figure 3F). They were sustainably released from each surface with an uptrend. It can be seen that the release features of these ions were consistent with the coating EDS analysis (Figure 3C). Previously, we found the optimal concentration of Zn for osteogenic capacity (Lin et al., 2013). Thus, the samples were prepared with fixed Zn concentration, approximately 6 wt% and total releasing profiles approximately 12 μg/cm^2^ after 14 days. Different doses of Sr were added to investigate the synergistic effect of Sr in implant osseointegration. The total releasing amounts of Sr in 1SrZn@TiO_2_, 2SrZn@TiO_2_, and 3SrZn@TiO_2_ are approximately 18 μg/cm^2^, 10 μg/cm^2^, and 4 μg/cm^2^ after 14 days. The highest releasing concentration in the 3SrZn@TiO_2_ group is approximately 0.02 mM far below 5 mM, the overdose of Sr screened in HUVECs system, which indicates biosafety of our Sr-modified implants. The releasing amounts of Ca and P are lower in Zn and/or Sr modified titanium samples compared to the TiO_2_ control group. We thus speculate that the dual elements, Zn and Sr, jointly improve the titanium implant on osteogenic poverty with angiogenesis as well as antibacterial ability.

### The Osteogenic Potential of Ovariectomized Bone Mesenchymal Cells on the Sr-Zn-Augmented Titanium Surfaces and the Vasotropic Effects of Strontium

**FIGURE 4:**
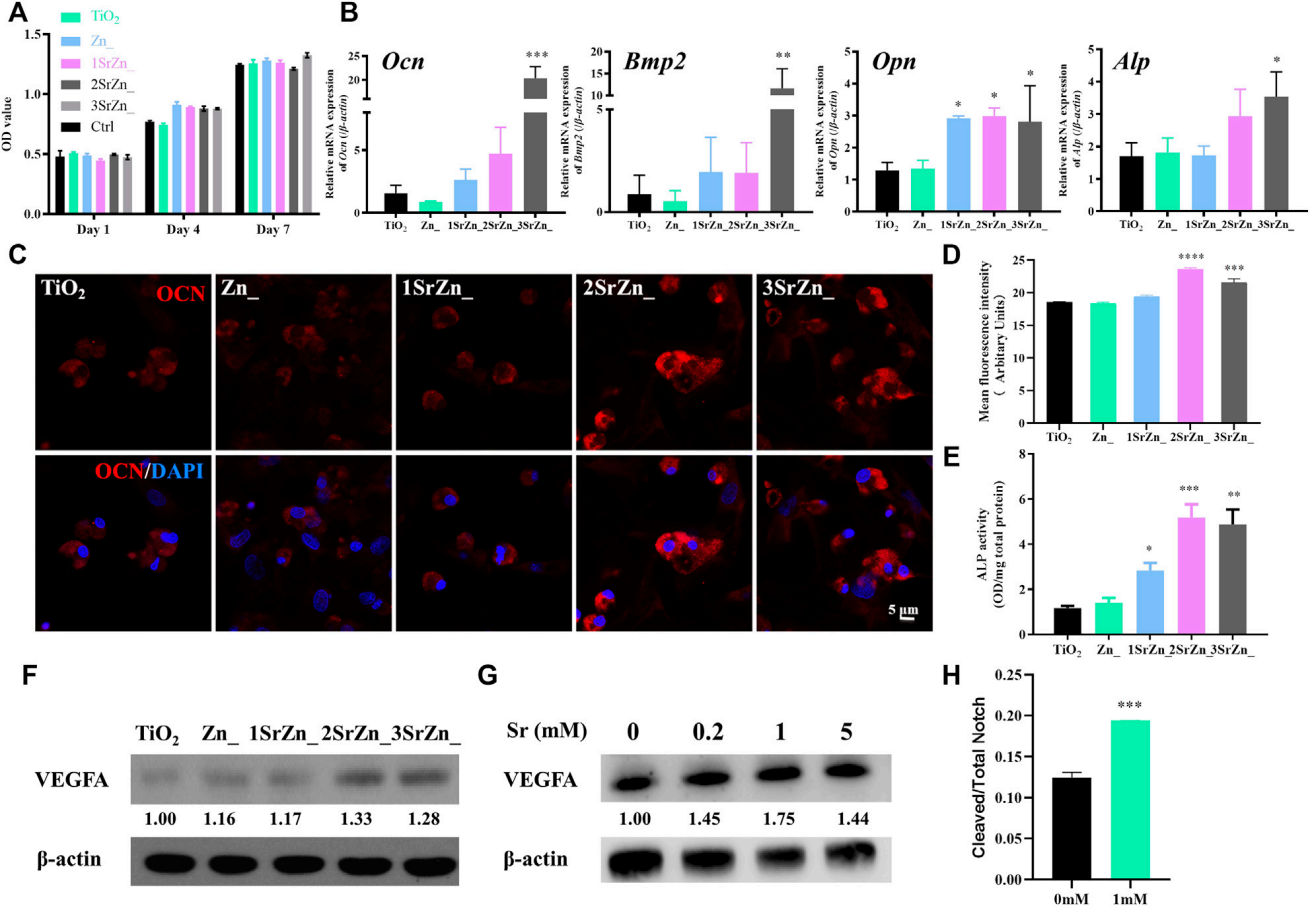
The osteogenic potential of OVX BMSCs on the Sr-Zn-augmented titanium surfaces and the vasotropic effects of Sr. **(A)** Cell proliferation activity assay of the OVX BMSCs on different samples evaluated by CCK-8, with culture medium containing no sample as control. **(B)** Temporal expression of osteogenic-related genes of OVX BMSCs on different surfaces at 7 days, including *Ocn*, *Opn*, *Bmp2*, and *Alp*. **(C)** Immunofluorescence assay of OCN expression in OVX BMSCs incubated on the coatings with an analysis of **(D)** mean fluorescence intensity. **(E)** ALP activity test of OVX BMSCs on surfaces. **(F)** VEGFA expression of OVX BMSCs seeded on samples for 7 days was tested by Western blot with quantification. **(G)** Western blot detection and analysis of VEGFA expression in HUVECs treated with 0, 0.2, 1, and 5 mM Sr for 7 days. **(H)** The ratio of cleaved and total Notch of HUVECs after 1 h in 1 mM Sr-containing medium and control medium by ELISA (*n* = 3, *p < 0.05, **p < 0.01, ***p < 0.001, ****p < 0.0001, compared with the TiO_2_ group or 0 mM).

Biological behaviors of BMSCs on an implant surface influence the success of early osseointegration. The contact bone formation on the implant surface initiates when osteoblasts adhere to the implant surface, cluster, and secrete bone matrix. However, inadequate bone formation by osteoblasts originating from BMSCs is a major reason for osteoporosis, which fails to compensate bone resorption. Impaired capability of proliferation and osteogenic differentiation in osteoporosis should be recovered around the implant site for better osseointegration at an early stage. In this study, OVX BMSCs are incubated on the samples to mimic peri-implant bone tissue spread and adhesion *in vitro*. The number of adhesive cells on the surface is increasing throughout the period, which indicates that the modified surface possesses good biocompatibility and is favorable for cell adherence (Figure 4A). In addition, the proliferation rates of the cells on each sample surface exhibit no significant difference after culturing for 7 days, suggesting that the surface chemistry with different combination of ions scarcely affect the proliferation ability of OVX BMSCs (Figure 4A). Enhancing osteoblastic differentiation of BMSCs has also been found in the groups containing Sr. Real-time PCR was performed to analyze the temporal expression of osteogenic genes of OVX BMSCs on different surfaces, including *Ocn*, *Opn*, *Bmp2*, and *Alp*, which are highly expressed on surfaces containing Sr (Figure 4B). The 3SrZn@TiO_2_ group shows the highest expression of *Bmp2* and *Alp* genes compared to other groups with significance (Figure 4B). OCN is a marker of osteoblastic differentiation that is specifically expressed in osteoblasts. Immunofluorescence was conducted to test the expression of OCN in OVX BMSCs. As exhibited in Figure 4C, OVX BMSCs adhering to 2SrZn@TiO_2_ and 3SrZn@TiO_2_ groups express much more OCN at 4 days than the other groups. Mean fluorescence intensity of immunofluorescence of OCN was measured in Figure 4D with the strongest expression in the 2SrZn@TiO_2_ group. The changes in the osteogenic differentiation were also evaluated by ALP activity test. The modified surface shows improvement after strontium substitution, and 2SrZn@TiO_2_ shows the highest level (Figure 4E). The positive effects of Sr on OVX BMSCs agree with previous studies that Sr induces higher expression of osteoblastic genes with increased bone nodules, resulting in improved bone mineral density, compensating for osteoporosis. Therefore, strontium-modified titanium surfaces in this study exhibit effective osteoinductive ability that includes the recruitment of immature cells and promote osteogenic differentiation, which involves normal bone healing process and is responsible for the majority of newly formed bone.

To clarify the regulation of strontium on angiogenesis and osteogenesis, the expression of VEGFA of BMSCs and HUVECs was detected. VEGFA is currently known to be an important factor involved in osteogenesis and angiogenesis coupling (Ramasamy et al., 2014). Apart from provoking the migration and proliferation of ECs, VEGFA is capable of indirectly stimulating osteogenesis through promoting autocrine angiogenesis (Diomede et al., 2020). In the current study, VEGF expression of OVX BMSCs seeded on surfaces is upregulated in a Sr dose-dependent manner at day 7 (Figure 4F). Osteoblast-derived VEGF is an important regulator of cell fate and bone formation in bone development and postnatal bone homeostasis (Hu and Olsen, 2016). Upregulation of osteogenic VEGF expression in this study indicates that the appropriate concentration of Sr establishes an angiogenic microenvironment and contributes to osteogenesis *via* angiogenic factors. Meanwhile, Sr promotes the protein expression of VEGFA of HUVECs with 1 mM at peak level (Figure 4G). Recent studies have revealed Notch signaling pathway functions a lot in the coupling of angiogenesis and osteogenesis of osteoporosis (Ramasamy et al., 2014; Luo et al., 2019). The ratio of cleaved Notch1 to total Notch1 significantly increased under the stimulation of 1 mM Sr on HUVECs (Figure 4H), which suggests activation of Notch signaling by Sr administration. VEGFA mediates the expression of Notch ligand Delta-like 4 (Dll4) and then activates the Notch signaling. Previous studies have proved that pharmacological reversal of the low Notch signaling may exert therapeutic benefit in osteoporotic patients by promoting angiogenesis-dependent bone formation (Fu et al., 2020). It is supposed in this study that Sr promotes the secretion of VEGFA from both HUVECs and BMSCs and activates the Notch signaling pathway of HUVECs for further angiogenic–osteogenic process, which awaits further evidence.

### The Antibacterial Ability of the Sr-Zn-Augmented Titanium Surfaces

**FIGURE 5:**
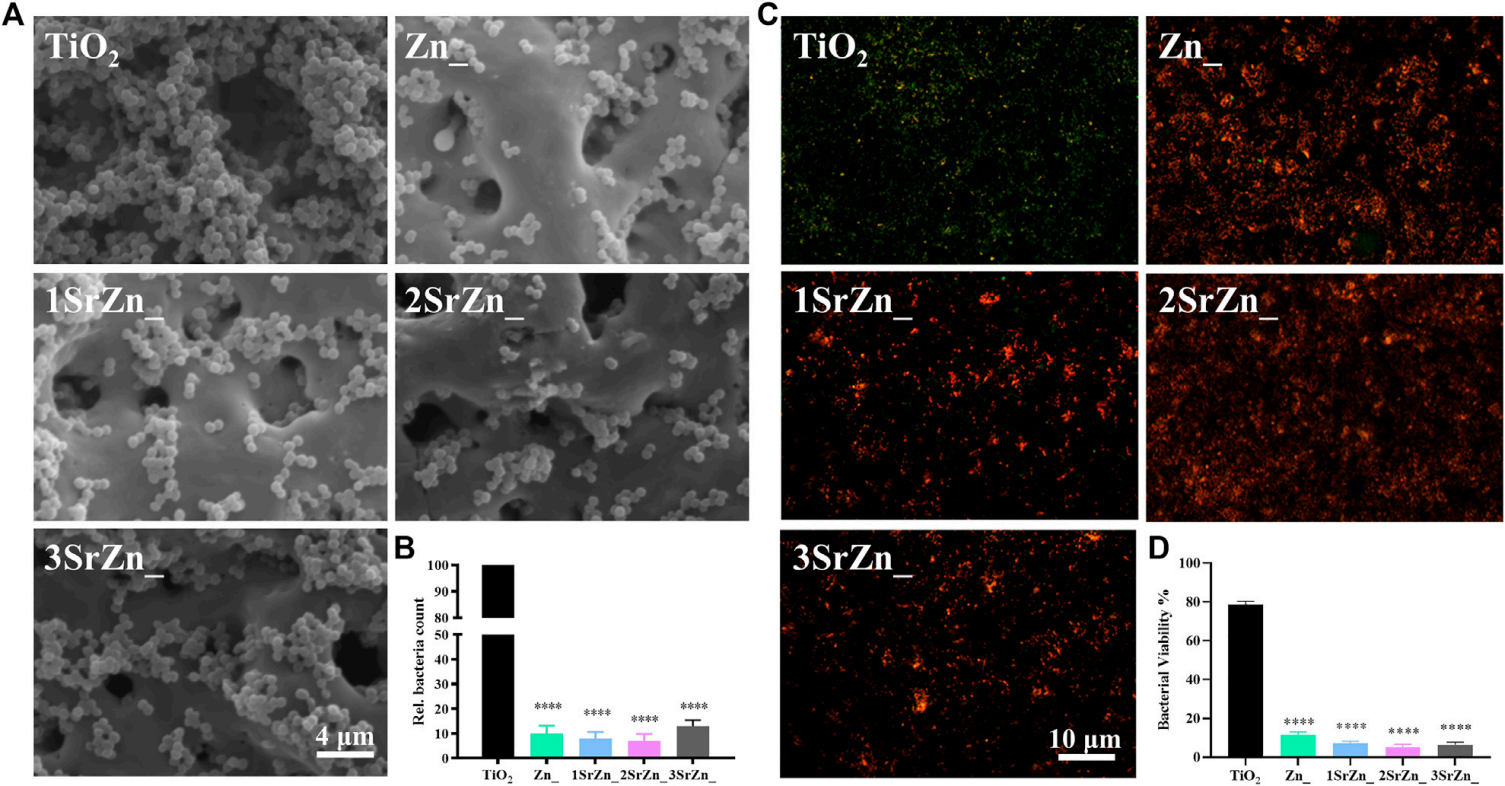
The antibacterial ability of the Sr-Zn-augmented titanium surfaces. **(A)** SEM images of MRSA on the sample surfaces after 24 h of incubation. **(B)** The numbers of MRSA on the sample surfaces counted through spread plate evaluation with the bacteria solution dissociated from surface and then cultivated on standard agar culture medium at 37°C for 24 h. **(C)** Immunofluorescence assay of Live/Dead of *P. gingivalis* on different titanium surfaces (Live bacteria, green; Dead bacteria, red), and **(D)** their quantitative results compared with the TiO_2_ group.

It is difficult to establish stable osseointegration in implant-associated infections as the bacteria form a biofilm on the prosthetic material (Van Dijk et al., 2020). Metal ions or nanoparticles are believed to be promising additives in developing antibacterial biomaterials, owing to possessing favorable bactericidal effects against antibiotic-resistant bacteria (Liu et al., 2019b). As implant-associated infections threaten the stable osseointegration, the antibacterial ability of these samples was evaluated. From the SEM of bacteria on various samples by using the stand strain MRSA, all the zinc-doped coatings exhibited good antibacterial ability, with significantly reduced bacteria remaining on the surface compared to the pure TiO_2_ coating with lots of adhering bacteria (Figure 5A). According to the bacterial counting results of the spread plate evaluation, zinc-doped coatings possessed an antibacterial ratio of approximately 90% relative to the control TiO_2_ coating (Figure 5B). The antibacterial abilities were measured and turned out almost the same on the four Zn-doped groups, namely, Zn@TiO_2_, 1SrZn@TiO_2_, 2SrZn@TiO_2_, and 3SrZn@TiO_2_. *P. gingivalis* is one of the major pathogenic bacteria of periimplantitis (Li et al., 2020). The bacterial Live/Dead staining of *P. gingivalis* on titanium surfaces was also conducted (Figure 5C). The four Zn-doped groups exhibit lower bacterial viability (Figure 5D). The results of spread plate evaluation and bacterial viability assay do not show related significant differences in Zn-doped groups with or without Sr, indicating that the antibacterial ability is mainly exerted by Zn but not Sr.

### Assessment of *in vivo* Osseointegration of Sr-Zn-Augmented Titanium Implant in Ovariectomized Rat Model

Biomaterial implants need to integrate into the host tissue without fibrous capsule and with certain mechanical properties. Evaluation of bone regeneration at the bone tissue/implant interface in particular, is essential for the clinical success of an implant (Palmquist, 2018; Guglielmotti et al., 2019). Based on the *in vitro* assessments of Sr-Zn-augmented titanium samples, the interface between bone and implant was evaluated in an OVX rat model in terms of micro-CT, biomechanical performance, and histological analysis.

**FIGURE 6:**
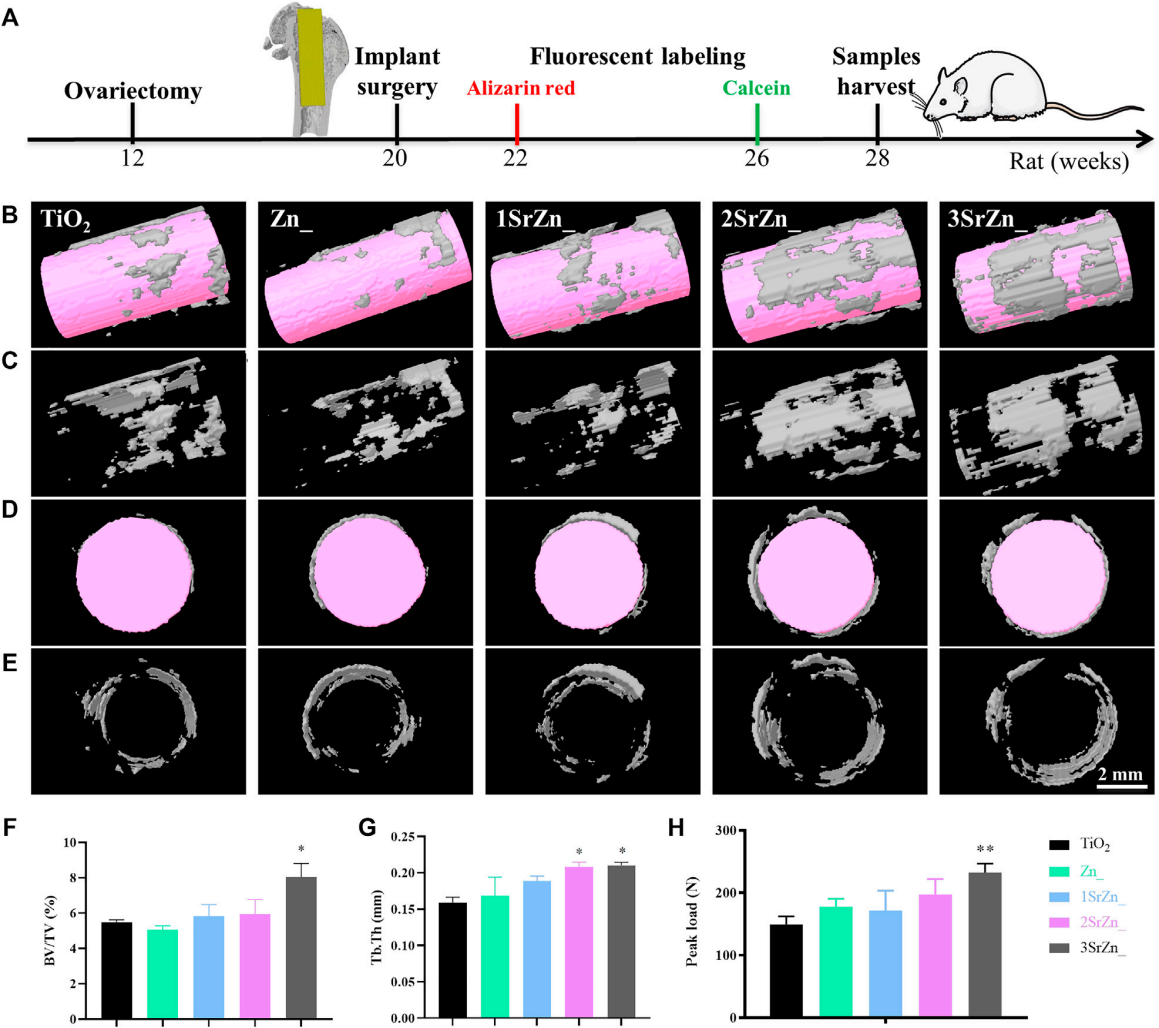
Quality and quantity of regenerated bone tissue on Sr-Zn-augmented titanium surfaces in the OVX rat model. **(A)** The scheme of animal operation: the 12-week-old rat femoral model was constructed by ovariectomy, and at 20 weeks old, the implant surgery was conducted. At 2 and 6 weeks after implant surgery, the fluorescent labeling was injected. The bone tissue and samples were harvested at 28 weeks old. **(B-E)** Reconstructed 3D models of surrounding bones in micro-CT with inserted implants of longitudinal section with **(B)** or without implant **(C)** and of transverse section with **(D)** or without implant **(E)**. Quantitative analysis includes **(F)** BV/TV and **(G)** Tb. Th. **(H)** Peak load of the samples compared with the TiO_2_ group.

Following the scheme in Figure 6A, the five groups of titanium rod were inserted to the femur bones of OVX rats for 8 weeks. To observe the newly formed bone tissue around the implants, 3D models of surrounding bones with inserted implants in the OVX rat model were reconstructed. The white part indicates the newly formed bone and the pink referred to the implants. The most obvious bone formation was observed in the 2SrZn@TiO_2_ and 3SrZn@TiO_2_ groups with high structure thickness and better bone contact (Figures 6B–E) compared with other samples in OVX rats. Furthermore, the quantitative micro-CT analysis shows that the BV/TV value (Figure 6F) and Tb.Th (Figure 6G) in the 2SrZn@TiO_2_ and 3SrZn@TiO_2_ groups were higher than those of the control group. In the test of biomechanical performance, the load-bearing ability test of the interface was conducted to evaluate the strength of bone-modified surface contact during bone repair (Figure 6H). The 3SrZn@TiO_2_ group showed the highest peak load. The 2SrZn@TiO_2_ and 3SrZn@TiO_2_ groups exhibit similar excellent performance in promoting bone regeneration *in vivo*, which indicates that variance in Sr concentration is not the only determinant of osteogenic effects. The complicated implant microenvironment and the synergistic effects of trace elements should be taken into further consideration. These data in the current study prove that the Sr-modified titanium implant improves the quality and quantity of regenerated bone tissue in OVX rats with better biomechanical performance.

**FIGURE 7:**
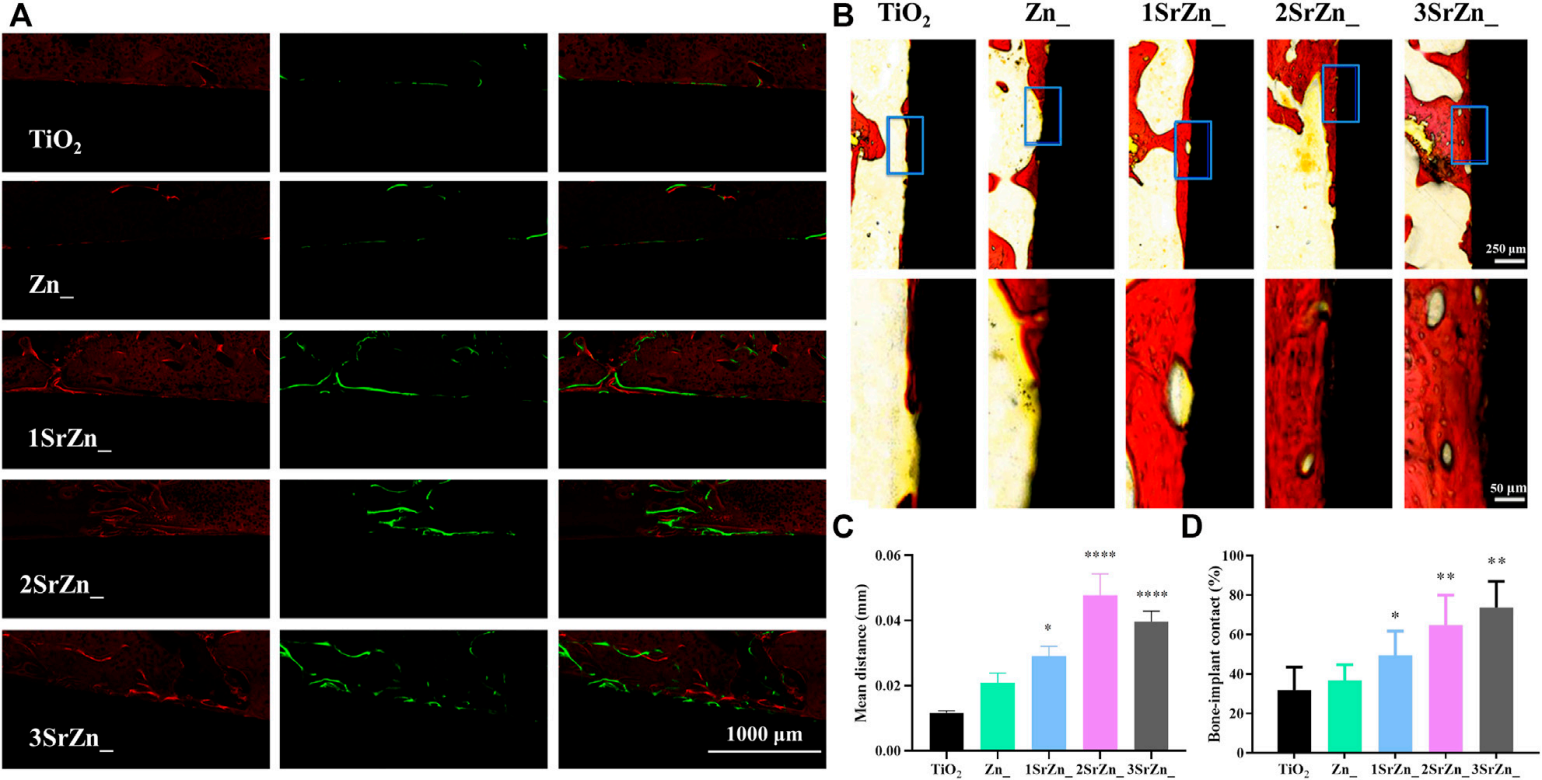
*In vivo* osseointegration. **(A)** Bone formation and deposition around the implant samples by polychrome sequential fluorescent labeling method; listed from left to right: alizarin red labeling (red) at 2 weeks and calcein (green) at 6 weeks after surgery of bone tissue and the merged image. **(B)** Van Gieson’s staining of the sections from rats at 8 weeks after implant surgery. **(C)** The mean distance between the two labeling lines of alizarin red and calcein (red and green) in **(A)** was calculated by ImageJ compared with the TiO2 group.**(D)** Quantification of the bone implant contact (BIC) level of the staining in **(B)** compared with the TiO2 group.

To further analyze the integration between implants and bone tissue, histological analysis was conducted in this study. In order to assess the bone formation and deposition around the implant, alizarin red and calcein were injected into the abdomens of rats at 2 and 6 weeks after implant surgery, respectively. Figure 7A evinces obvious fluorescent bone deposition close to the implant surface in Sr-modified groups. The mean distance between alizarin red and calcein presents the amount of bone formation in 4 weeks as shown in Figure 7C. Sr-modified groups display larger mean distance, suggesting highly active bone formation under the effects of Sr, especially in 2SrZn@TiO_2_ and 3SrZn@TiO_2_. Van Gieson staining is one of the histological tests to investigate bone formation for the examination of osseointegration. All of the Sr-modified samples exhibit increased direct bone–implant contact based on more bone formation, suggesting better osseointegration than the TiO_2_ group and Zn@TiO_2_ group in osteoporotic bone (Figure 7B). The newly formed bone exhibits vessel-like structures (Figure 7B), which may be the implication of the angiogenic potential of Sr modified into the implant. The BIC was calculated in Figure 7D based on Van Gieson staining, indicating the best contact of osseointegration presenting in the 2SrZn@TiO_2_ group and 3SrZn@TiO_2_ group. López-Valverde et al. reviewed that titanium implants doped with strontium showed significantly higher BIC, and the coating also improved the implants’ biomechanical properties (Lopez-Valverde et al., 2019). The BIC level of 3SrZn@TiO_2_ was nearly 80% (Figure 7D) where little fibrous or connective tissue prevents direct contact with bone (Figure 7B), which may be the reason why the 3SrZn@TiO_2_ group performed best in a push-out test. With the measurement of micro-CT, biomechanical, and histological difference, the 2SrZn@TiO_2_ and 3SrZn@TiO_2_ groups are suitable for the prediction of a direct structural and functional connection between living bone and the surface of a load-carrying implant.

Our above results indicate that strontium contributes to the contact of osseointegration. The effects of strontium on osseointegration have been proven in previous studies. Liu et al. indicated that micro/nano strontium-loaded surface implants enhance bone–implant osseointegration *in vivo* (Liu et al., 2019a). In addition, Sr possesses anti-adipogenesis capability and is expected to be applied in dental implantation in the aged population for rapid osseointegration (Zhou et al., 2019). In our study, Sr improves the angiogenic potential of ECs and regulates the osteogenesis by promoting the angiogenic factor VEGFA secreted by ECs and BMSCs. Therefore, Sr creates an angiogenic microenvironment in osteoporotic bone favoring osseointegration around implants. Strontium-modified titanium implants are thus expected to be a promising option for clinical implants in osteoporotic patients.

## Conclusion

Together with all the outcomes, we can speculate that the combination doping of Zn and Sr should be one of the candidate modification methods to optimize the titanium implant’s properties in osteoporotic bone microenvironment with promoted osteogenesis and angiogenesis. Apart from the conventional studying spot in osteogenesis, Sr plays an attractive role in the coupling of osteogenesis and angiogenesis, as demonstrated in a co-culture system with HUVECs and hBMSCs. Assessment of effects of bioactive ions of different doses under a multi-system mimicking an implanting microenvironment brings about more comprehensive evaluation of the effective dose of ions, which provides clues to prediction of biofunction *in vivo* with higher accuracy. As further exploration on the function and mechanism of bioactive factors involved in implanting sites is under way, it is a promising prospect to develop bioactive multifunctional implant surfaces for clinical application.

## Data Availability

The raw data supporting the conclusion of this article will be made available by the authors, without undue reservation.
