# Supplementary material for: Trace Element-Augmented Titanium Implant With Targeted Angiogenesis and Enhanced Osseointegration in Osteoporotic Rats
Source: Front Chem. 2022 Feb 17;10:839062. doi: 10.3389/fchem.2022.839062 (PMC8902677; doi:10.3389/fchem.2022.839062)
Supplement: Supplementary file 1 [file Table1.DOCX]

Appendix Table Primers for real-time polymerase chain reaction (PCR).

| **Gene** | **Prime sequence**  **(F, forward; R, reverse)** | **Product size (bp)** | **Accession number** |
| --- | --- | --- | --- |
| **β-Actin** | F: AGGGAGTGATGGTTGGAATG  R: GATGATGCCGTGTTCTATCG | 107 | NM_031004.2 |
| **OCN** | F: GCCCTGACTGCATTCTGCCTCT  R: TCACCACCTTACTGCCCTCCTG | 158 | NM_013414.1 |
| **OPN**  **BMP-2**  **ALP**  **VEGFA**  **Ang-1**  **Gapdh** | F: CAAGCGTGGAAACACACAGCC  R: GGCTTTGGAACTCGCCTGACTG  F: ATGGGTTTGTGGTGGAAGTG  R: TGTTTGTGGAGTGGATGTC  F: GTCCCACAAGAGCCCACAAT  R: CAACGGCAGAGCCAGGAAT  F: TTGAGTTGGGAGGAGGATGT  R: TGGCAGGCAAACAGACTTC  F: TGCCATTACCAGTCAGAGGC  R: AGCACCGTGTAAGATCAGGC  F: GACCTGACCTGCCGTCTA  R: AGGAGTGGGTGTCGCTGT | 165  167  172  115  181  148 | NM_012881.2  NM_017178.1  NM_013059.1  NM_001110333.1  NM_001146.5  NM_001357943.2 |
